# 2676. Development of the World's First Automated Phenotypic Antibiotic Susceptibility Test System for *Neisseria gonnorrhoeae*

**DOI:** 10.1093/ofid/ofad500.2287

**Published:** 2023-11-27

**Authors:** Mitchell D Newton, Sachidevi Puttaswamy, Andrew Isakson, Jared McPeake, Ted Hadfield, Roy Swiger

**Affiliations:** Acenxion Biosystem, Kansas City, Kansas; Acenxion Biosystems, Kansas City, Kansas; Acenxion Biosystems, Kansas City, Kansas; Acenxion Biosysystems, Kansas City, Kansas; Acenxion Biosystems, Kansas City, Kansas; Acenxion Biosystems, Kansas City, Kansas

## Abstract

**Background:**

With the increasing prevalence of antibiotic resistance of *Neisseria gonorrhoeae* (*NG*), clinical reliance on empiric treatment is no longer an option. Providing clinicians with fast, accurate, susceptibility results for directed patient treatment of *NG* cannot be ignored anymore. Here we describe the development of the world’s first phenotypic automated Antibiotic Susceptibility Test system specific to *NG*. The system is reliable, seamlessly integrates into existing sample workflows, and provides full customization. This yields a user-friendly device for rapid and actionable patient treatment.

**Methods:**

The system consists of Hardware; Software & Firmware; and Consumables that are single-use disposable. The system allows the growth of fastidious *Neisseria gonorrhoeae* while continuously monitoring for growth or lack thereof in the presence of antibiotics in real time, displaying reports via a user-friendly interface. The samples tested include QC strains and challenge strains of *N. gonorrhoeae* along with select isolates across multiple antibiotics of relevance at multiple concentrations. All testing has been done in parallel with the phenotypic broth dilution method for verification in-house and/or compared against expected or published results.

**Results:**

The results of the analysis of gonococcal strains are generated in as little as four (4) hours and are based on phenotypic and broth dilution methodology. We show (a) results from analysis of quality control strains, and CDC/FDA Antibiotic Resistance (AR) Bank and (b) a demonstration of easy integration using clinical laboratory sample workflow. In 100% of cases, our method produced a clear selection of the MIC and corresponding S/I/R interpretations allowing for calculated Categorical Agreement.

**Conclusion:**

This data demonstrates not merely the feasibility but realizability of an automated, phenotypic AST for *NG*. The results here demonstrate that it is possible to make informed treatment decisions for cases of gonorrhea.

**Disclosures:**

**All Authors**: No reported disclosures

